# Controlling Nano-to-Microscale Multilevel Architecture in Polymeric Microfibers through Polymerization-Induced Spontaneous Phase Separation

**DOI:** 10.3390/polym15112537

**Published:** 2023-05-31

**Authors:** Maya Molco, Amir Keilin, Adira Lunken, Shiran Ziv Sharabani, Mark Chkhaidze, Nicole Edelstein-Pardo, Tomer Reuveni, Amit Sitt

**Affiliations:** 1 School of Chemistry, Faculty of Exact Sciences, Tel Aviv University, Tel Aviv 6997801, Israel; 2 The Center for Nanoscience and Nanotechnology, Tel Aviv University, Tel Aviv 6997801, Israel; 3 The Center for Physics & Chemistry of Living Systems, Tel Aviv University, Tel Aviv 6997801, Israel

**Keywords:** hierarchical structures, nanospheres, jeffamine/glutaraldehyde reaction, electrospun core–sheath fibers, microreactors, ouzo effect

## Abstract

Hierarchically structured polymeric fibers, composed of structural nanoscale motifs that assemble into a microscale fiber are frequently found in natural fibers including cellulose and silk. The creation of synthetic fibers with nano-to-microscale hierarchical structures represents a promising avenue for the development of novel fabrics with distinctive physical, chemical, and mechanical characteristics. In this work, we introduce a novel approach for creating polyamine-based core–sheath microfibers with controlled hierarchical architectures. This approach involves a polymerization-induced spontaneous phase separation and subsequent chemical fixation. Through the use of various polyamines, the phase separation process can be manipulated to produce fibers with diverse porous core architectures, ranging from densely packed nanospheres to segmented “bamboo-stem” morphology. Moreover, the nitrogen-rich surface of the core enables both the chemisorption of heavy metals and the physisorption of proteins and enzymes. Our method offers a new set of tools for the production of polymeric fibers with novel hierarchical morphologies, which has a high potential for a wide range of applications such as filtering, separation, and catalysis.

## 1. Introduction

The ongoing advances in the ability to control the physical, chemical, and mechanical properties of polymeric fibers, and the possibility to incorporate them with new technologies [1], enables the creation of fabrics with new features and properties including functional fibers which can adsorb moisture [2], conduct electricity [3,4], and have biomedical applications [5]. Performance fabrics can exhibit high mechanical durability [6], self-cleaning [7,8], anti-fouling [9], and even self-healing properties [10]. There are smart fabrics that can sense their environment and change their features accordingly [11]. While many of the properties described above emanate from the chemical structure of the polymer, in recent years it has become evident that the geometrical architecture of the fiber, and in particular the combination of micro- and nanoscale hierarchical geometrical features, plays a major role in determining the physical, mechanical, and sometimes even the chemical properties of the fiber [12].

The hierarchical geometrical structure and morphology are also highly significant when considering the inner structure of polymeric fibers. The inner structure of the fiber can have a major effect on a range of factors including the mechanical performance, wettability, porosity, and chemical reactivity of the fiber [13]. The formation of a complex hierarchical inner structure within the fiber, which combines different dimensions and scales, offers a large specific area and many heterogeneous interfaces [14,15,16,17,18]. Such a hierarchical fiber structure is a common feature in many natural systems [19,20], and is also appealing for a range of applications including fluid transport, separation and filtering, drug delivery and encapsulation, tissue growth, energy storage, and catalysis [21,22,23,24]. However, affecting the internal geometry is not trivial, as this part of the fiber is not exposed to the environment, and hence its structure must be induced in the fabrication step, or rely on internal processes, including phase separation or self-assembly, that can alter the morphology of the inner domain of the fiber post-fabrication.

One of the most versatile methods for obtaining fibers in the nano-to-microscale is electrospinning. In this approach, a polymer solution (or a melt) is dispensed through a capillary, while a high voltage is applied between the capillary and a collecting conductive surface. This results in the ejection of a fluid jet that is stretched and thinned as it solidifies, forming fibers on the collecting surface [25,26,27]. Electrospinning provides a range of methods for controlling the inner architecture of the system including electrohydrodynamic coaxial and side-by-side spinning [26,28], coaxial spinning with sacrificial fillers [29], emulsion electrospinning [30], and post-fabrication in situ chemical and physical modification [31,32].

Core–sheath electrospinning is one of the most common approaches for constructing complex fiber architectures. In a typical core–sheath electrospinning process, the formation of a solid sheath, mediated by rapid solvent evaporation from the surface of the fiber, occurs first and precedes the solidification of the core. This provides a period in which the core components are still in a dynamic liquid form that is confined within the walls of the solid sheath. Reaching the final configuration of the core is much slower and is strongly affected by the liquid attributes of the core at this stage, and in particular the liquid–solid surface tension between the core and the solid sheath [28,32,33,34]. While in most cases the slow solidification of the core leads to a standard core–sheath structure, a combination of strong adhesion between the core and the sheath, accompanied by a large volume reduction of the core, can lead to the formation of hollow fibers [33].

In this work, we introduce a new approach to the fabrication of highly porous core–sheath microfibers and microcylinders with versatile hierarchical core architectures. The fibers, which are made of a polyamine core embedded in a poly-lactic-co-glycolic acid (PLGA) sheath, are collected over a gel matrix that contains glutaraldehyde (GA), a highly potent crosslinker and fixation agent for amine moieties. Upon penetration of the GA into the core, chemically induced spontaneous phase separation and chemical fixation occur simultaneously. The nature of the phase separation, which depends on the polyamine used and the spatial confinement, dictates the obtained core morphology, which can range from densely packed nano-spheres to periodically segmented longitudinal compartments. The nitrogen-rich environment of the core allows the spatial-selective binding of different species to the core compartment, including the chemisorption of metal ions, as well as the physisorption of enzymes. This, together with the highly porous morphology of the core and its high specific surface area, paves the path for potential applications of such fiber in a range of technological applications, including separation, filtering, and catalysis.

## 2. Experimental

### 2.1. Materials

Poly lactic-co-glycolic acid (PLGA) (lactide to glycolide ratio = 85:15; Mw = 50–75 kDa), polyethylene glycol Mw ~1,000,000 Da (PEG-1 MDa), polyethylene glycol Mw ~5,000,000 Da (PEG-5 MDa), trimethylolpropane tris[poly(propylene glycol), amine terminated] ether (Jeffamine T-403), 3,3,5-trimethylhexamethylene-diamine (Jeffamine D-230), tetraethylenepentamine (TEPA), poly(propylene glycol) bis(2-aminopropyl ether) average Mn ~2000 Da (Jeffamine D-2000), chloroform, tetrahydrofuran (THF), dimethylformamide (DMF), visualizing markers for CLSM poly[(m-phenylenevinylene)-alt-(2,5-dihexyloxy-p-phenylenevinylene)] (blue), fluorescein isothiocyanate (FITC), Tween 20, glutaraldehyde (GA) (50% in water solution), catalase from bovine liver (lyophilized powder, 2000–5000 units/mg protein) and ruthenium(III) chloride hydrate were all purchased from Sigma-Aldrich (St. Louis, MO, USA). Cryosectioning medium, optimal cutting temperature (OCT) gel (Tissue-plus OCT compound, Fisher Healthcare, Waltham, MA, USA) was purchased from Fisher Scientific (Waltham, MA, USA). Nitric acid (70%), hydrochloric acid (36%), and hydrogen peroxide (30%) were all purchased from BioLab (Toronto, ON, Canada). Sodium phosphate dibasic heptahydrate and sodium phosphate monobasic monohydrate were purchased from J.T. Baker (Phillipsburg, NJ, USA). All the materials were used as bought, without further purification. Phosphate buffer (PB) (0.1 M, pH 6) was prepared by dissolving 3.67 g of sodium phosphate dibasic heptahydrate and 11.91 g of sodium phosphate monobasic monohydrate in 1 L of water.

### 2.2. Spontaneous Phase Separation in Solutions

The phase separation and fixation that occurred during the reaction between the polyamine and the GA were examined using a light microscope. A droplet of polyamine (Jeffamine T-403/Jeffamine D-2000/TEPA) was placed on a glass slide. Next, a droplet of 50% GA in water solution was slowly injected onto the glass slide in proximity to the polyamine droplet, until the two droplets came into contact. As a control, the process was repeated with polyamine and DI water droplets. The interface between the two solutions was monitored and examined microscopically using an inverted bright-field microscope.

### 2.3. Jetting Solutions

For all the solutions described below, the concentrations are given in the polymer’s mass to solvent’s volume (in g/mL). PLGA solution for the sheath was prepared by dissolving 0.400 g PLGA in a mixture of 0.500 mL THF and 0.500 mL DMF (1:1 *v*/*v*). A trace amount of the blue visualizing marker was added to the solution. A jetting solution of PEG/polyamine for the core was prepared by dissolving 0.025 g PEG (Mw ~1 MDa), 0.025 g PEG (Mw ~5 MDa), and 0.500 g polyamine (either Jeffamine T-403, Jeffamine D-230, Jeffamine D-2000, or TEPA) in 1.000 mL chloroform. OCT/GA gel was prepared by mixing OCT with a solution of GA in deionized water (50%) in a ratio of 3:1 *v*/*v*.

### 2.4. Fiber Electrospinning

The experimental setup contained two syringe pumps (New Era), a power supply (DC voltage source, Gamma High Voltage Research, Ormond Beach, FL, USA), and a rotating drum collector. The PLGA and PEG/polyamine solutions were dispensed via a metallic coaxial core (23-gauge)–sheath (14-gauge) needle (Ramé-Hart, Succasunna, NJ, USA). Both the core and the sheath solutions were dispensed at a constant flow rate of 0.150 mL/h. A driving voltage of 1.5–4 kV resulted in a stable jet and the core–sheath fibers were collected over a collecting drum covered with a homogeneous layer of OCT/GA gel with a thickness of ~2 mm, at a tip-to-ground distance of 14 cm and a drum rotating speed of 60 rpm.

### 2.5. Cryosectioning

The OCT/GA layer containing the jetted fibers was dried while rotating overnight. Next, the dried OCT sample was embedded into OCT gel in a cryomold. The embedded sample was frozen and cryo-sectioned using a cryostat (Leica CM3050 S, Leica, Wetzlar, Germany). The cryo-sectioned fibers in the OCT gel were then suspended in Tween 20 solution (0.01% *v/v* in water) and the OCT was dissolved by a gentle rocking of the sample overnight. The sectioned fibers were separated from residuals of OCT and GA gel by five successive steps of washing and centrifugation. The clean sectioned fibers were kept in a known volume of Tween 20 solution (0.01% *v/v* in water) and their concentration was calculated using a hemocytometer (in particles per mL units). The analysis of the sectioned fibers was performed using a scanning electron microscope (SEM) (Zeiss GeminiSEM 300, Zeiss, Jena, Germany) in a high vacuum (WD ~5 mm; 2–3 kV), and by confocal microscopy (Olympus IX83, Olympus, Tokyo, Japan).

### 2.6. Metalions Adsorption

The chemisorption of metallic ions to the microcylinders was conducted and analyzed in the following procedure: 200 µL of microcylinders, with a concentration of 3.50 × 10^5^ microcylinders per mL, were gently mixed overnight with 1 mL of 0.1 M metal ions solution. The microcylinders were then washed with Tween 20 solution (0.01% *v/v* in water) several times to remove unbound ions.

The spatial distribution of the adsorbed metal ions along the particles was examined using energy dispersive spectroscopy (EDS) (X-Flash 6/60, Bruker, Billerica, MA, USA). The average loading of metal per particle was obtained using inductively coupled plasma mass spectrometry (ICP-MS) (ICP-MS 7800, Agilent, Santa Clara, CA, USA). For this measurement, a sample of microcylinders with adsorbed metal ions was dried under a vacuum overnight and then weighed. A mixture of water, nitric acid (70%), hydrochloric acid (36%), and hydrogen peroxide (30%) in a ratio of 2:1:3:1 was added to the sample. The sample was digested in a microwave oven which heated the sample from RT to 180 °C for 20 min and was kept at 180° C for 35 min, followed by a cooling process to RT. Next, the sample was diluted to a 50 mL total volume with water and further diluted 1000 times with 2% nitric acid. The mass percentage of the metal in the sample was measured.

### 2.7. Enzyme Immobilization and Activity Testing

Catalase was physisorbed to the microcylinders in the following procedure: 200 µL of microcylinders with a concentration of 3.00 × 10^5^ microcylinders per mL were gently mixed overnight with 300 µL of enzyme solution (~1.5 mg/mL in a pH 6 phosphate buffer) and 500 µL of pH 6 phosphate buffer. The microcylinders were then washed with Tween 20 solution (0.01% *v/v* in water) several times to remove the unbound enzyme. The activity of the immobilized catalase was examined using a light microscope. A droplet of the catalase-immobilized microcylinders was placed on a glass substrate. Next, a droplet of Tween 20 (1% *v/v* in water) was placed on top of the microtubes and then another droplet containing the enzyme’s substrate, hydrogen peroxide (~1.5% *v/v* in water), was added. The enzyme decomposed the hydrogen peroxide to water and oxygen gas, and oxygen bubbles were ejected from the edges of the microcylinders.

## 3. Results and Discussion

While the external architecture of a fiber is exposed and can be manipulated in a range of chemical, physical, and even mechanical means, a realization of a hierarchical inner structure within fibers is much more complex and calls for an approach that will spontaneously induce the desired morphology and architecture in a bottom-up manner. In this work, we utilize the coupling of spontaneous self-emulsification to a rapid chemical crosslinking that arrests the phase-separated morphology, as a novel approach for constructing microscale fibers with complex hierarchical inner structures.

**Figure 1:**
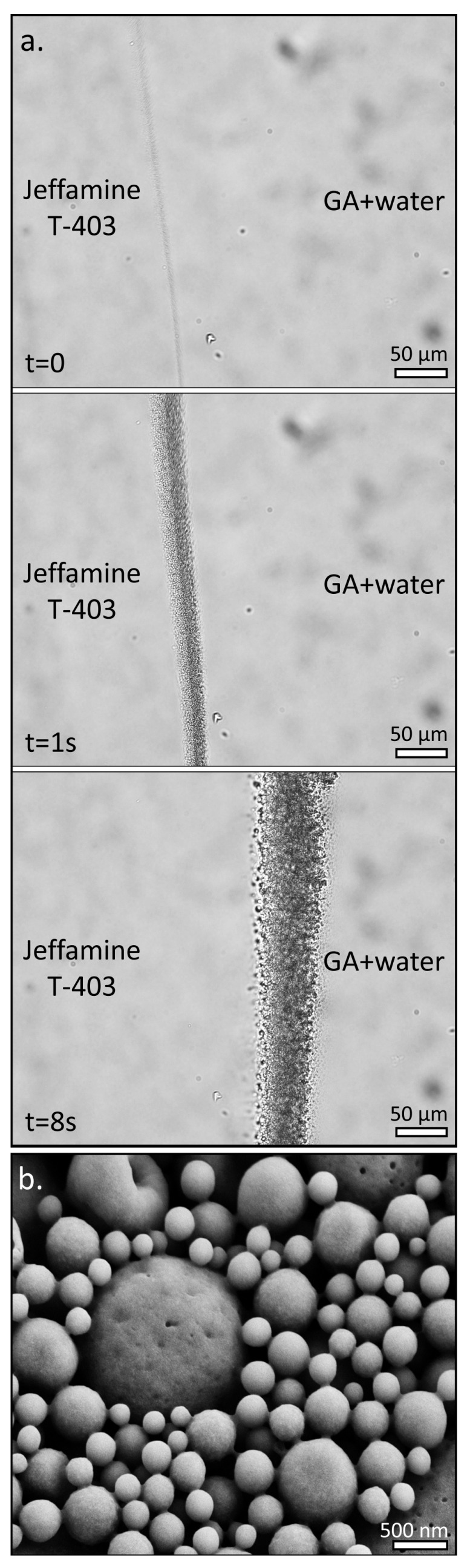
(**a**) Time lapse of the spontaneous emulsification process of Jeffamine T-403 (Left Phase) and GA in water (50%) (Right Phase), showing the formation of spontaneous emulsification at the interface between two droplets of the liquids. (**b**) SEM image of the resulting residue, showing the formation of nano- and microspheres.

To achieve this task, we chose a system that consists of the polyamine trimethylolpropane tris[poly(propylene glycol), amine terminated] ether (Jeffamine T-403) and glutaraldehyde (GA) in water, taking advantage of the high reactivity of GA with amine groups. Figure 1a and Movie S1 demonstrate the self-emulsification process, also known as the “Ouzo Effect” [35,36], at the interface of a droplet of Jeffamine T-403 and a droplet of GA in water, taken using bright-field microscopy. Upon the formation of an interface, the Jeffamine T-403 phase penetrates the GA/water phase, and microdroplets are vigorously produced. The emulsification process is accompanied by the crosslinking of the Jeffamine T-403 molecules by their reaction with GA. The reaction between the Jeffamine T-403 and GA molecules results in the formation of a highly crosslinked polymeric network. While the exact binding scheme of the GA with amines is still not fully understood, it can lead to a range of nitrogen-containing species including amines, imines, and even nitrogen-containing heterocyclic groups [37]. Regardless, the reaction leads to a strong and wide emission band (Figure S1), indicative of the formation of a conjugated system [38,39]. Interestingly, Jeffamine T-403 is miscible with water (Movie S2), hence we believe that crosslinking also plays a major role in the emulsification process through the formation of the Jeffamine T-403-GA polymeric network, which is less soluble in water and leads to phase separation. As the reaction progresses, the formed Jeffamine T-403-GA polymeric network droplets are fully crosslinked into solid nano- and microspheres, clearly seen when the residue is examined in the scanning electron microscope (SEM) (Figure 1b). It should be noted, that unlike many cases in which the emulsification precedes the polymerization [40,41,42,43], we believe that here polymerization is a crucial step towards spontaneous emulsification.

**Figure 2:**
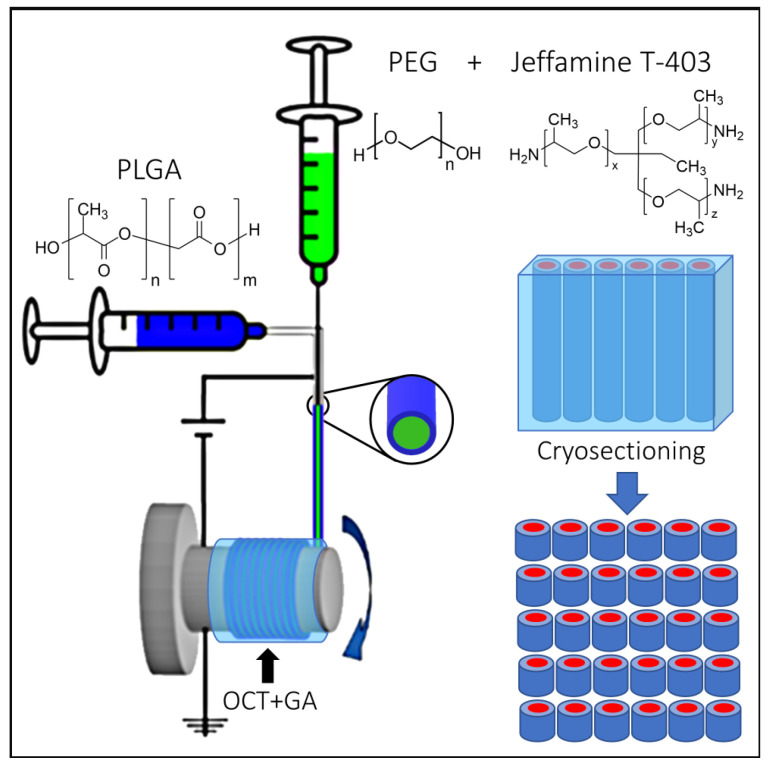
A schematic illustration of the fabrication process. The core solution (in green) contains PEG + Jeffamine T-403, and the shell solution (in blue) contains PLGA. The two solutions are injected simultaneously to achieve a core−sheath structure and are collected on a rotating drum covered with OCT and GA (**left**). After the fabrication of the fibers, they are cryo-sectioned into microcylinders with a defined length (**right**).

Utilizing this phase separation and solidification scheme inside the polymeric fibers requires maintaining the phases in their liquid form without losing the structural attributes of the fiber. To achieve this, we used a core–sheath architecture in which the sheath solution is composed of hydrophobic poly lactic-co-glycolic acid (PLGA) dissolved in a mixture of tetrahydrofuran (THF) and dimethylformamide (DMF) (1:1 volumetric ratio). The core is composed of Jeffamine T-403, polyethylene glycol Mw ~5,000,000 Da (PEG-5 MDa), and polyethylene glycol Mw ~1,000,000 Da (PEG-1 MDa) (20:1:1 by weight) dissolved in chloroform. The PEG significantly increases the viscosity of the solution, and the entanglement of its chains provides a polymeric scaffold that also pulls the relatively small Jeffamine T-403 molecules. The two solutions were jetted simultaneously through a co-axial metallic needle in a core–sheath configuration. When voltage was applied between the needle and the collector, the system formed a stable jet, and fibers with a core–sheath configuration, dictated by the structure of the needle, were formed. The fabrication process is schematically depicted in Figure 2.

To introduce the GA, optimal cutting temperature (OCT) gel was mixed with a solution of 50% GA in water in a volume ratio of 3:1 immediately before use. The relatively viscous gel was evenly smeared over the rotating drum, and the fibers were collected over the rotating drum in an aligned fashion along the direction of the rotation and embedded in the gel. Already a few minutes after the embedding of the fibers in the OCT/GA gel, their color changed from colorless to red, indicative of the reaction between the Jeffamine T-403 and the GA. The OCT gel, composed of water-soluble glycols and resins, was allowed to dry on the rotator overnight, forming a thin dry film embedded with the fibers. The dry OCT film containing the aligned fibers was either dissolved in water to release the intact fibers or further embedded in OCT, frozen, and cryo-sectioned. The sectioned samples were then immersed in a Tween 20 solution (0.1% *v/v* in water) to remove the excess OCT through repeated washing and centrifugation cycles, resulting in clean microcylinders with well-defined lengths.

**Figure 3:**
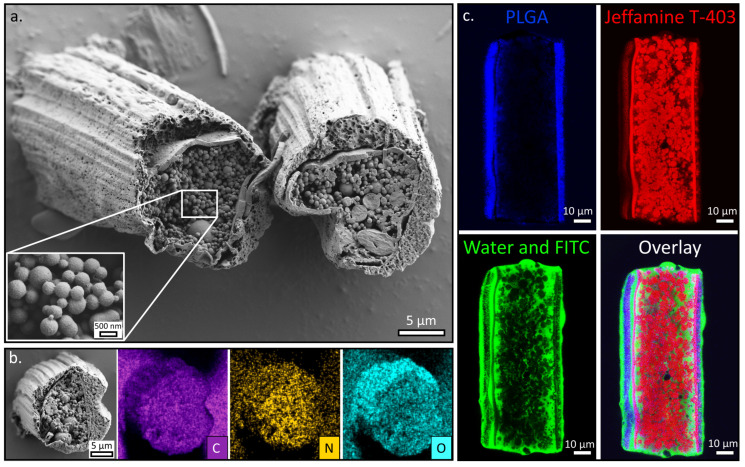
(**a**) SEM micrograph of typical cryo-sectioned “pixie-stick” fibers showing the outer (PLGA) and inner (PEG + Jeffamine T-403) sheaths and the nano- and microspheres at the core. Inset shows a magnification of the nanospheres. (**b**) EDS elemental analysis of a sectioned fiber showing excess nitrogen in the core (in yellow) and oxygen in the sheath (cyan), while carbon is homogeneously distributed in the entire fiber (purple). (**c**) CSLM cross-sections along a representative sectioned fiber immersed in water with green-emitting FITC. The outer PLGA sheath is depicted in blue (**top left**), the crosslinked Jeffamine T-403-GA microspheres and the inner sheath in red (**top right**), the water in green (**bottom left**), and the overlay (**bottom right**).

Figure 3a shows a typical SEM image of microcylinders obtained from a cryo-sectioned fiber. The obtained microcylinders exhibit a “pixie stick” architecture that is composed of a dual-layered sheath filled with densely packed nano- and microspheres. The mean outer diameter of the fibers is 16.3 ± 1.9 µm, while the mean inner diameter is 12.1 ± 1.8 µm (Figure S2). The spheres can be divided into two populations: the majority (90%) of the spheres are in the nanoscale range and exhibit a Gaussian size distribution with a mean diameter of 475 ± 157 nm; 10% of the spheres have a diameter in the range of 1.0–2.5 µm, with a mean diameter of 1660 ± 398 nm (Figure S3).

Energy dispersive spectroscopy (EDS) elemental analysis of the fiber’s cross-section (Figure 3b) shows that there is a higher concentration of nitrogen in the center of the fiber and the inner sheath, while a higher concentration of oxygen is observed in the outer sheath. As expected, carbon is homogeneously distributed in the fiber. This confirms that the spheres are composed of Jeffamine T-403, which is the only nitrogen-containing species in the system, while the outer sheath, composed mainly of PLGA, contains a high concentration of oxygen. It should be noted that the EDS is not quantitative because the samples were produced and handled in an atmospheric environment and, therefore, they absorb nitrogen and oxygen from the air.

To further characterize the composition and morphology of the fibers, we used confocal laser scanning microscopy (CLSM) imaging. To identify the compartment containing PLGA, a blue-emitting polymeric fluorophore was added to the PLGA solutions before the electrospinning process. As mentioned above, the product of the Jeffamine T-403–GA crosslinking exhibited strong and wide emission, with a peak at a wavelength of 561 nm, and was traced in the red channel. To examine the porosity between the spheres in the core and the percolation, the system was immersed in water that contained green-emitting fluorescein isothiocyanate (FITC), which was traced in the green channel. The fibers were subsequently imaged, and the spatial distributions of the different fluorophores were obtained (Figure 3c). The CLSM image reveals the existence of an outer sheath composed of PLGA (in blue) and an inner sheath that is made of Jeffamine T-403/PEG (in red). The nano- and microspheres can be seen within the inner sheath (in red). Examining the percolation of water (in green) indicates that most of the voids around the spheres are interconnected and can be accessed by the water. Furthermore, in some cases, a gap exists between the inner and outer sheaths, which is also observed in the SEM, and the water can penetrate this gap as well.

To examine the effect of the concentration of GA in the OCT gel on the formation of the hierarchical structure, four different GA concentrations in the gel were tested: no GA, 1%, 6%, and 12%. When no GA was used, the core solution did not solidify, and hence upon sectioning and washing, tubular particles were obtained (Figure S5A). In the case of 1% GA, tubular microparticles were formed, with spheres covering mostly the luminal walls of the tubes (Figure S5B). This shows that the emulsification and crosslinking of the core occur first at the core–sheath interface, thus indicating that the process evolves via a slow penetration of the GA through the shell towards the center of the core. At higher concentrations, the obtained microcylinders were filled with nano- and microspheres, indicating a full reaction of the core with the GA (Figure S5C,D).

Based on these results, we propose the following scheme for the formation of the “pixie stick” architecture. As the fibers are formed, initially the outer PLGA sheath solidifies, accompanied by the formation of an inner sheath of PEG with traces of Jeffamine T-403, in accordance with the process described before for PEG/polyester systems [32,33]. A liquid Jeffamine-rich phase remained in the core. Once the fiber is immersed in the GA-containing gel, GA slowly penetrates the core either by diffusion or through small cracks in the solid PLGA sheath. As the GA penetrates the fiber, it first reacts with the PEG-Jeffamine T-403 sheath and further solidifies it, but also leads to its shrinking, which induces the formation of a well-defined boundary and separation from the outer PLGA shell. As the GA continues to penetrate, it induces spontaneous emulsification in the liquid Jeffamine T-403 core, resulting in the formation of the spheres.

In this scheme, the gel-collecting medium has several roles. Most trivially, it acts as a large reservoir for the GA. The embedding of the fibers in the gel also decreases the evaporation rate of the solvents, thus keeping the core in a liquid phase and the shell prone to diffusion. Lastly, it acts as a stress-relieving scaffold that eliminates interfacial stresses, which occur in the fiber/collector and fiber/air interfaces, and thus preserves the circular cross-section of the fiber despite its soft inner core. Interestingly, introducing the GA/water phase directly on top of dry fibers of similar composition collected on a bare substrate resulted in fibers with a standard core–sheath architecture with a slightly oblate cross-section, and the penetration of the GA and its reaction with the Jeffamine T-403, indicated by the appearance of a red color, took several days instead of a few minutes in the case of a collection into the gel.

**Figure 4:**
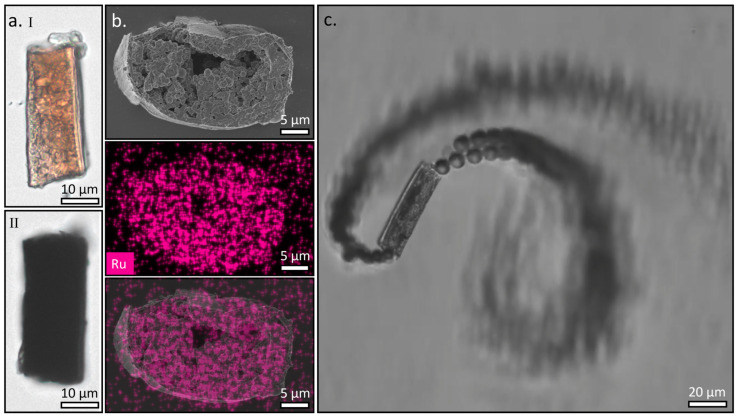
(**a**) Bright-field micrograph of a crosslinked Jeffamine T-403 containing microcylinder before (**I**), and after (**II**) immersion in a solution of 0.1 M RuCl_3_ in water. The color of the microparticle became much darker, indicating the adsorption of Ru^3+^ ions. (**b**) SEM image of the cross-section of a microcylinder after adsorption of Ru^3+^ (top), the corresponding EDS mapping of ruthenium (middle), and an overlay of the two (bottom). (**c**) Bright-field micrograph of a catalase-modified crosslinked Jeffamine T-403 microcylinder upon immersion in hydrogen peroxide solution (1.5% in water). Jets of bubbles emerge from both sides of the microcylinder, indicating that the disproportionation reaction is catalyzed selectively inside the cylinder.

The high porosity of the Jeffamine T-403 fibers makes them excellent candidates for the adsorption of different species, especially because of the nitrogen-rich chemistry of the core. For example, imine and pyridine groups can form complexes with transition metals. To examine the capability to utilize the system for metal adsorption, we used ruthenium as a case study because it has a high tendency to form complexes with a range of nitrogen-containing species [44,45]. The sectioned fibers were added to a solution of the metal ions (RuCl_3_, 0.1 M) and incubated overnight. Next, the solution was repeatedly centrifuged and washed with Tween 20 solution (0.1% *v/v* in water) to remove the excess ions that were not chemisorbed to the microparticles. After incubation with the ruthenium, a clear change in the color of the particles was observed, changing from orange to black (Figure 4a(I,II)). The adsorption of the ruthenium was examined in EDS to identify the position of the adsorbed metal, and the traces of ruthenium were mainly identified in the core of the fiber (Figure 4a(III)). The amount of ruthenium adsorbed per mass of particles was analytically determined using inductively coupled plasma mass spectrometry (ICP-MS), indicating 10% ruthenium by weight. Given a particle’s average length of 50 µm and an outer diameter of 16 µm, the amount of ruthenium adsorbed per length is 6.8 µg/m (the mass per volume is 33 mg/mL). The full analysis is summarized in the Supplementary Material Section S6.

The nitrogen-rich environment is also an effective agent for selective physisorption and the non-covalent binding of proteins and enzymes. To explore this, we examined the binding of the enzyme catalase, which catalyzes the disproportionation of hydrogen peroxide (H_2_O_2_) to water and oxygen. The microcylinders were incubated with a solution of catalase in a phosphate buffer (PB), followed by the removal of the excess unattached enzyme by successive washing and centrifuging cycles until the supernatant showed no enzymatic activity. Upon immersing the cleaned microcylinders in a solution of 1.5% hydrogen peroxide in water, a significant ejection of oxygen bubbles from the edges of microcylinders was observed, accompanied by a circular motion of the microcylinders in the water (Figure 4b and Movie S3). Bright-field microscopy examination shows that the formation of the oxygen bubbles is initiated inside the core of the microcylinders, and the bubbles propagate towards the edges, where they are excreted out of the particle. The regioselectivity of the catalysis indicates the selective adsorption of the enzyme to the nanospheres located at the core, and the high reactivity even at low H_2_O_2_ concentrations shows the high binding efficiency to the core.

**Figure 5:**
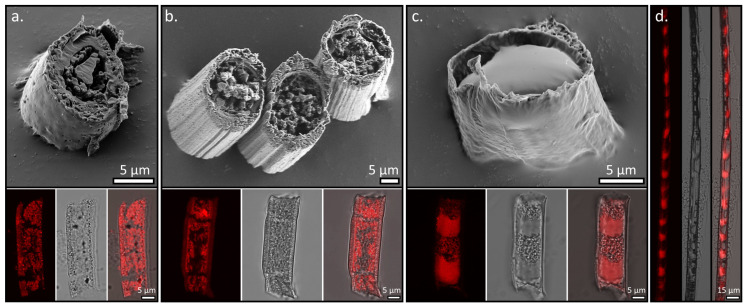
Cryo-sectioned fibers containing (**a**) TEPA, (**b**) Jeffamine D-230, and (**c**) Jeffamine D-2000. Lower panels show for each fiber (from left to right) a CLSM cross-section of the different fibers (crosslinked multi-amine emission is depicted in red), the bright-field micrograph, and the overlap of the two. (**d**) The CLSM cross-section of an entire crosslinked Jeffamine D-2000 fiber (**left**), its bright-field micrograph (**middle**), and an overlay of the two (**right**).

Finally, we examined the extension of this approach to other polyamines by replacing Jeffamine T-403 with other polyamines including tetraethylenepentamine (TEPA), 3,3,5-trimethylhexamethylene-diamine (Jeffamine D-230), and poly (propylene glycol) bis (2-aminopropyl ether), with an average Mn ~2000 Da (Jeffamine D-2000). In the case of TEPA, which is smaller than Jeffamine T-403 and has five amine sites, for the bulk solutions we observed a similar phase separation process as for Jeffamine T-403, yet the spontaneous emulsification process was much slower (Figure S6 and Movie S4). SEM and CLSM images of the obtained fibers indicate that the core forms a coral-shaped morphology made of a combination of longitudinal thin pillars and spheres (Figure 5a). In the case of Jeffamine D-230, which has a high chemical resemblance to Jeffamine T-403, but only two amine sites, the obtained morphology resembles that of Jeffamine T-403, but the spheres were less smooth and the overall density of the spheres in the core is lower, probably due to less efficient crosslinking (Figure 5b).

In the case of Jeffamine D-2000, which has a much larger molar weight and hence a significantly lower solubility in water, the bulk phase separation process substantially differed from that of the small polyamine molecules (Figure S7 and Movie S5). In this case, the two phases formed a clear interface, and a reverse emulsion of water in Jeffamine D-2000 was obtained through the rapid formation of water microdroplets at the interface, which penetrated the Jeffamine D-2000 phase. Over time, the microscale water droplets condensed and solidified together to form a solid bulk-crosslinked phase within the Jeffamine D-2000 phase.

The clear phase separation of the two phases was also observed in the fibers with Jeffamine D-2000 in the core. However, when combined with the confinement of the phases in the core, this led to a unique segmented morphology that resembles a bamboo stem, where the core separates to distinguished longitudinal solid segments of crosslinked Jeffamine D-2000, separated from each other by voids, as can be seen in a cross-section of the fibers obtained through confocal imaging (Figure 5c,d). Analysis of the confocal images indicates that the segments and voids are of similar lengths of ~16 µm (Supplementary Material Section S4).

This unique segmented morphology highly resembles the fragmentation of surface-deposited polymeric fibers upon drying, and hence we believe the two systems share similarities in the segmentation mechanism [46]. Such segmentation is a result of two opposing stresses that are formed in the fiber throughout the solidification stage. In our case, the opposing forces occur throughout the phase separation and solidification processes: the crosslinking of the Jeffamine D-2000 leads to the shrinking of the core phase and to a reduction of its volume, while the interfacial adhesion of the Jeffamine D-2000 solution to the sheath, induced by the confinement, opposes the shrinking process. The combination of these two opposing forces, together with the phase separation process, results in higher shrinking in the middle of the core with respect to the core–sheath interface, and the difference in shrinking between the interface and the center of the core leads to a buildup of internal stress in the core. While direct observation of the segmentation process, which might have led to a better understanding of the process, cannot be facilitated because the fibers are immersed in the gel and cannot be visualized throughout the process, a strong indication for this mechanism can be obtained from SEM images of the sectioned fibers and from confocal images (Figure 5c,d), which show that the edges of the solid segment across the fiber are relatively concave.

## 4. Conclusions

In this work, we present a new approach that combines the spontaneous phase separation of polyamines conjugated with chemical fixation as a synthetic route for the formation of polymeric core–sheath fibers with a controlled hierarchical multilevel structure. By changing the polyamine molecules, different phase separation schemes can be achieved and fibers with diverse porous core architectures, including densely packed nanospheres, coral-like, and segmented morphologies, can be obtained. The nitrogen-rich surface of the core was demonstrated to enable the chemisorption of the heavy metal ruthenium, as well as the physisorption of proteins and enzymes, making it a potent system for filtering and sensing applications. The obtained results demonstrate the engineering of the core of electrospun polymeric fibers through in situ chemical and physical processes that modify the architecture, and consequently the properties of the fibers. This is enabled by the collection of the fibers into a sacrificial gel matrix, which preserves the fiber’s contour and induces the chemical process. The scheme demonstrated here can be extended to other spontaneous emulsification systems, opening a path for more complex hierarchical fibers with new and exciting properties, and a high potential for a range of applications, including filtering and catalysis, in the near future.

## Data Availability

All data are included in the article.

## Supplementary Materials

The following supporting information can be downloaded at https://www.mdpi.com/article/10.3390/polym15112537/s1: The emission profile of amine-GA (Figure S1), size distribution of the outer and inner diameters of Jeffamine T-403 fibers (Figure S2), size distribution of the nano- and microspheres of Jeffamine T-403 fibers (Figure S3), size distribution of the Jeffamine D-2000 segments and the gaps between them in Jeffamine D-2000 fibers (Figure S4), effect of GA concentration on the core’s architecture (Figure S5), calculation of the mass of ruthenium adsorbed per length or volume units, phase separation in the bulk solutions of Tepa (Figure S6) and Jeffamine D-2000 (Figure S7) with GA (50% in water). (PDF), self-emulsification process at the interface of Jeffamine T-403 and GA droplets (Movie S1), solubility of Jeffamine T-403 in water at the interface of Jeffamine T-403 and water droplets (Movie S2), the disproportionation of hydrogen peroxide by immobilized catalase in the microcylinders (Movie S3), self-emulsification process at the interface of TEPA and GA droplets (Movie S4), and the self-emulsification process at the interface of Jeffamine D-2000 and GA droplets (Movie S5) (MP4). Calculation of the average concentration of ruthenium in a fiber (Table S1).

## References

[1] Jingcheng L., Reddy V.S., Jayathilaka W.A.D.M., Chinnappan A., Ramakrishna S., Ghosh R. (2021). Intelligent Polymers, Fibers and Applications. Polymers.

[2] Jia T., Wang Y., Dou Y., Li Y., Jung de Andrade M., Wang R., Fang S., Li J., Yu Z., Qiao R. (2019). Moisture Sensitive Smart Yarns and Textiles from Self-Balanced Silk Fiber Muscles. Adv. Funct. Mater..

[3] Halder O., Layani-Tzadka M.E., Ziv Sharabani S., Markovich G., Sitt A. (2022). Metal Nanowires Grown in Situ on Polymeric Fibres for Electronic Textiles. Nanoscale Adv..

[4] Zhu S., Wang M., Qiang Z., Song J., Wang Y., Fan Y., You Z., Liao Y., Zhu M., Ye C. (2021). Multi-Functional and Highly Conductive Textiles with Ultra-High Durability through ‘Green’ Fabrication Process. Chem. Eng. J..

[5] dos Santos D.M., Correa D.S., Medeiros E.S., Oliveira J.E., Mattoso L.H.C. (2020). Advances in Functional Polymer Nanofibers: From Spinning Fabrication Techniques to Recent Biomedical Applications. ACS Appl. Mater. Interfaces.

[6] Pu Y., Yang X., Zhang Y., Li L., Xie Y., He B., Yuan D., Ning X. (2020). Fabrication and Characterization of Highly Oriented Composite Nanofibers with Excellent Mechanical Strength and Thermal Stability. Macromol. Mater. Eng..

[7] Luo J., Wang L., Huang X., Li B., Guo Z., Song X., Lin L., Tang L.-C., Xue H., Gao J. (2019). Mechanically Durable, Highly Conductive, and Anticorrosive Composite Fabrics with Excellent Self-Cleaning Performance for High-Efficiency Electromagnetic Interference Shielding. ACS Appl. Mater. Interfaces.

[8] Dalawai S.P., Saad Aly M.A., Latthe S.S., Xing R., Sutar R.S., Nagappan S., Ha C.-S., Kumar Sadasivuni K., Liu S. (2020). Recent Advances in Durability of Superhydrophobic Self-Cleaning Technology: A Critical Review. Prog. Org. Coat..

[9] He Z., Bao B., Fan J., Wang W., Yu D. (2020). Photochromic Cotton Fabric Based on Microcapsule Technology with Anti-Fouling Properties. Colloids Surf. A.

[10] Shuai L., Guo Z.H., Zhang P., Wan J., Pu X., Wang Z.L. (2020). Stretchable, Self-Healing, Conductive Hydrogel Fibers for Strain Sensing and Triboelectric Energy-Harvesting Smart Textiles. Nano Energy.

[11] Singh A.V., Rahman A., Sudhir Kumar N.V.G., Aditi A.S., Galluzzi M., Bovio S., Barozzi S., Montani E., Parazzoli D. (2012). Bio-Inspired Approaches to Design Smart Fabrics. Mater. Des..

[12] Yang G., Li X., He Y., Ma J., Ni G., Zhou S. (2018). From Nano to Micro to Macro: Electrospun Hierarchically Structured Polymeric Fibers for Biomedical Applications. Prog. Polym. Sci..

[13] Badmus M., Liu J., Wang N., Radacsi N., Zhao Y. (2021). Hierarchically Electrospun Nanofibers and Their Applications: A Review. Nano Mater. Sci..

[14] Wu J., Wang N., Zhao Y., Jiang L. (2013). Electrospinning of Multilevel Structured Functional Micro-/Nanofibers and Their Applications. J. Mater. Chem. A.

[15] Wang C., Wang J., Zeng L., Qiao Z., Liu X., Liu H., Zhang J., Ding J. (2019). Fabrication of Electrospun Polymer Nanofibers with Diverse Morphologies. Molecules.

[16] Rezabeigi E., Wood-Adams P.M., Demarquette N.R. (2018). Complex Morphology Formation in Electrospinning of Binary and Ternary Poly(Lactic Acid) Solutions. Macromolecules.

[17] Huang C., Thomas N.L. (2018). Fabricating Porous Poly(Lactic Acid) Fibres via Electrospinning. Eur. Polym. J..

[18] Huang C., Thomas N.L. (2020). Fabrication of Porous Fibers via Electrospinning: Strategies and Applications. Polym. Rev..

[19] John M.J., Thomas S. (2008). Biofibres and Biocomposites. Carbohydr. Polym..

[20] Ramamoorthy S.K., Skrifvars M., Persson A. (2015). A Review of Natural Fibers Used in Biocomposites: Plant, Animal and Regenerated Cellulose Fibers. Polym. Rev..

[21] Peng N., Widjojo N., Sukitpaneenit P., Teoh M.M., Lipscomb G.G., Chung T.-S., Lai J.-Y. (2012). Evolution of Polymeric Hollow Fibers as Sustainable Technologies: Past, Present, and Future. Prog. Polym. Sci..

[22] Hu X., Liu S., Zhou G., Huang Y., Xie Z., Jing X. (2014). Electrospinning of Polymeric Nanofibers for Drug Delivery Applications. J. Control. Release.

[23] Pant B., Park M., Park S.-J. (2019). Drug Delivery Applications of Core-Sheath Nanofibers Prepared by Coaxial Electrospinning: A Review. Pharmaceutics.

[24] Liu R., Hou L., Yue G., Li H., Zhang J., Liu J., Miao B., Wang N., Bai J., Cui Z. (2022). Progress of Fabrication and Applications of Electrospun Hierarchically Porous Nanofibers. Adv. Fiber Mater..

[25] Ramakrishna S., Fujihara K., Teo W.-E., Lim T.-C., Ma Z. (2005). An Introduction to Electrospinning and Nanofibers.

[26] Roh K.-H., Martin D.C., Lahann J. (2005). Biphasic Janus Particles with Nanoscale Anisotropy. Nat. Mater..

[27] Lahann J. (2011). Recent Progress in Nano-Biotechnology: Compartmentalized Micro- and Nanoparticles via Electrohydrodynamic Co-Jetting. Small.

[28] Sun Z., Zussman E., Yarin A.L., Wendorff J.H., Greiner A. (2003). Compound Core–Shell Polymer Nanofibers by Co-Electrospinning. Adv. Mater..

[29] Gualandi C., Zucchelli A., Fernández Osorio M., Belcari J., Focarete M.L. (2013). Nanovascularization of Polymer Matrix: Generation of Nanochannels and Nanotubes by Sacrificial Electrospun Fibers. Nano Lett..

[30] Yarin A.L. (2011). Coaxial Electrospinning and Emulsion Electrospinning of Core-Shell Fibers. Polym. Adv. Technol..

[31] Molco M., Laye F., Samperio E., Ziv Sharabani S., Fourman V., Sherman D., Tsotsalas M., Wöll C., Lahann J., Sitt A. (2021). Performance Fabrics Obtained by In Situ Growth of Metal–Organic Frameworks in Electrospun Fibers. ACS Appl. Mater. Interfaces.

[32] Sitt A., Soukupova J., Miller D., Verdi D., Zboril R., Hess H., Lahann J. (2016). Microscale Rockets and Picoliter Containers Engineered from Electrospun Polymeric Microtubes. Small.

[33] Dror Y., Salalha W., Avrahami R., Zussman E., Yarin A.L., Dersch R., Greiner A., Wendorff J.H. (2007). One-Step Production of Polymeric Microtubes by Co-Electrospinning. Small.

[34] Vats S., Anyfantakis M., Honaker L.W., Basoli F., Lagerwall J.P.F. (2021). Stable Electrospinning of Core-Functionalized Coaxial Fibers Enabled by the Minimum-Energy Interface Given by Partial Core–Sheath Miscibility. Langmuir.

[35] Vitale S.A., Katz J.L. (2003). Liquid Droplet Dispersions Formed by Homogeneous Liquid-Liquid Nucleation: “The Ouzo Effect”. Langmuir.

[36] Vratsanos M.A., Xue W., Rosenmann N.D., Zarzar L.D., Gianneschi N.C. (2023). Ouzo Effect Examined at the Nanoscale via Direct Observation of Droplet Nucleation and Morphology. ACS Cent. Sci..

[37] Migneault I., Dartiguenave C., Bertrand M.J., Waldron K.C. (2004). Glutaraldehyde: Behavior in Aqueous Solution, Reaction with Proteins, and Application to Enzyme Crosslinking. Biotechniques.

[38] Ma X., Sun X., Hargrove D., Chen J., Song D., Dong Q., Lu X., Fan T.-H., Fu Y., Lei Y. (2016). A Biocompatible and Biodegradable Protein Hydrogel with Green and Red Autofluorescence: Preparation, Characterization and In Vivo Biodegradation Tracking and Modeling. Sci. Rep..

[39] Lee K., Choi S., Yang C., Wu H.-C., Yu J. (2013). Autofluorescence Generation and Elimination: A Lesson from Glutaraldehyde. Chem. Commun..

[40] Ganachaud F., Katz J.L. (2005). Nanoparticles and Nanocapsules Created Using the Ouzo Effect: Spontaneous Emulsification as an Alternative to Ultrasonic and High-Shear Devices. ChemPhysChem.

[41] Aschenbrenner E., Bley K., Koynov K., Makowski M., Kappl M., Landfester K., Weiss C.K. (2013). Using the Polymeric Ouzo Effect for the Preparation of Polysaccharide-Based Nanoparticles. Langmuir.

[42] Lepeltier E., Bourgaux C., Couvreur P. (2014). Nanoprecipitation and the “Ouzo Effect”: Application to Drug Delivery Devices. Adv. Drug Deliv. Rev..

[43] Kempe H., Kempe M. (2022). Ouzo Polymerization: A Bottom-up Green Synthesis of Polymer Nanoparticles by Free-Radical Polymerization of Monomers Spontaneously Nucleated by the Ouzo Effect; Application to Molecular Imprinting. J. Colloid Interface Sci..

[44] Wamba A.G.N., Kofa G.P., Koungou S.N., Thue P.S., Lima E.C., Dos Reis G.S., Kayem J.G. (2018). Grafting of Amine Functional Group on Silicate Based Material as Adsorbent for Water Purification: A Short Review. J. Environ. Chem. Eng..

[45] Otsuki J., Wu G., Kaneko R., Ebata Y., Mishra A.K., Mishra L. (2018). Orbital Tuning of Ruthenium Polyimine Complexes by Ligand Design: From Basic Principles to Applications. Ruthenium Chemistry.

[46] Edelstein-Pardo N., Molco M., Rathee P., Koren G., Tevet S., Ziv Sharabani S., Beck R., Amir R.J., Sitt A. (2022). Anisotropic Microparticles through Periodic Autofragmentation of Amphiphilic Triblock Copolymer Microfibers. Chem. Mater..

