# Supplementary material for: Controlling Nano-to-Microscale Multilevel Architecture in Polymeric Microfibers through Polymerization-Induced Spontaneous Phase Separation
Source: Polymers (Basel). 2023 May 31;15(11):2537. doi: 10.3390/polym15112537 (PMC10255670; doi:10.3390/polym15112537)
Supplement: Supplementary file 1 [file polymers-15-02537-s001.zip › SI.pdf]

Supporting information

Controlling Nano-to-Microscale Multilevel Architecture in Polymeric Microfibers through Polymerization-Induced Spontaneous Phase Separation

Maya Molco[1,2,3], Amir Keilin[1], Adira Lunken[1], Shiran Ziv Sharabani[1,2,3], Mark Chkhaidze[1], Nicole Edelstein-Pardo[1,2,3], Tomer Reuveni[1,2], Amit Sitt[1,2,3]

1. School of Chemistry, Faculty of Exact Sciences, Tel-Aviv University, Tel-Aviv 6997801, Israel

2. Tel Aviv University Center for Nanoscience and Nanotechnology, Tel-Aviv University, Tel-Aviv 6997801, Israel

3. The Center for Physics & Chemistry of Living Systems, Tel Aviv University, Tel-Aviv 6997801, Israel

Corresponding Author

* Amit Sitt, School of Chemistry, Tel Aviv University, Tel Aviv, Israel, amitsitt@tauex.tau.ac.il

S1. Emission profile of amine-GA

The crosslinking of the amine moieties with glutaraldehyde (GA) leads to the formation of a wide and asymmetric emission peak with a maximum at a wavelength of ~560 nm (**Figure S1**). The emission was collected using the confocal microscope under excitation wavelength of 405 nm. While the exact emitting species is unclear, it is attributed to the formation of highly conjugated imine groups that are formed in the reaction between the amine groups in the polyamine and the GA.

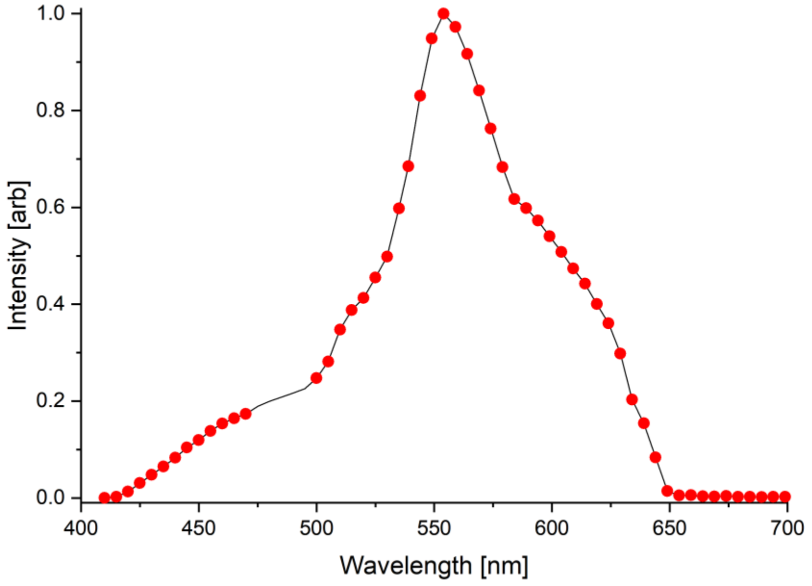

Figure S1: Emission profile of the crosslinked Jeffamine T-403 excited at 405 nm. Red dots indicate the measured intensities, and the black line is guidance to the eye.

S2. Size distribution of the outer and inner diameters of Jeffamine T-403 fibers

The outer and inner diameters of the Jeffamine T-403 crosslinked fibers were measured from SEM micrographs of the cross-section of cryo-sectioned fibers. Due to the fiber's elliptical-like shape, two inner and two outer perpendicular diameters of 60 fibers were measured and the average diameters of each fiber was calculated. The outer diameter is referred to the diameter of the entire fiber, edge to edge, and the inner diameter includes the PEG rich outer core and the spheres inside. The average outer diameter is 16.3±1.9 µm (**Figure S2A**) and the average inner diameter is 12.1±1.8 µm (**Figure S2B**).

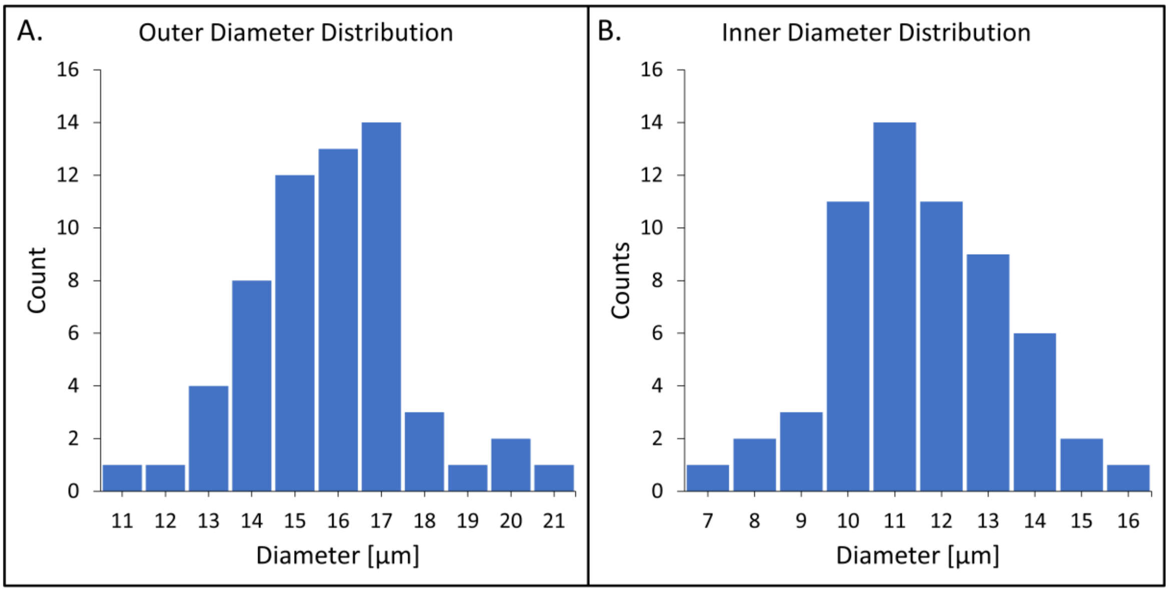

Figure S2: Size distribution of the **A.** inner and **B.** outer diameters of the crosslinked Jeffamine T-403 microcylinders.

S3: Size distribution of the nano and microspheres of Jeffamine T-403 fibers

The diameters of the nano and microspheres formed inside the Jeffamine T-403 crosslinked fibers were also measured from SEM micrographs of the cross-section of cryo-sectioned fibers. To obtain the size distribution of the spheres, one hundred spheres were measured. While this is only a representative population in the cross-sections of the fibers, we believe it provides a good indication to the size distribution throughout the entire fiber. While the distribution is relatively wide, we can clearly observe two populations: The majority of the spheres (90%) are in the nanoscale range and exhibit a Gaussian size distribution with a mean diameter of 475±157 nm; 10% of the spheres have a diameter in the microscale range (1-2.5 µm), with a mean diameter of 1660±398 µm (**Figure S3**). It should be noted that even though the number of microspheres is smaller, they occupy a significantly larger volume, and hence have a major effect on the porosity and on the total surface area of the sample.

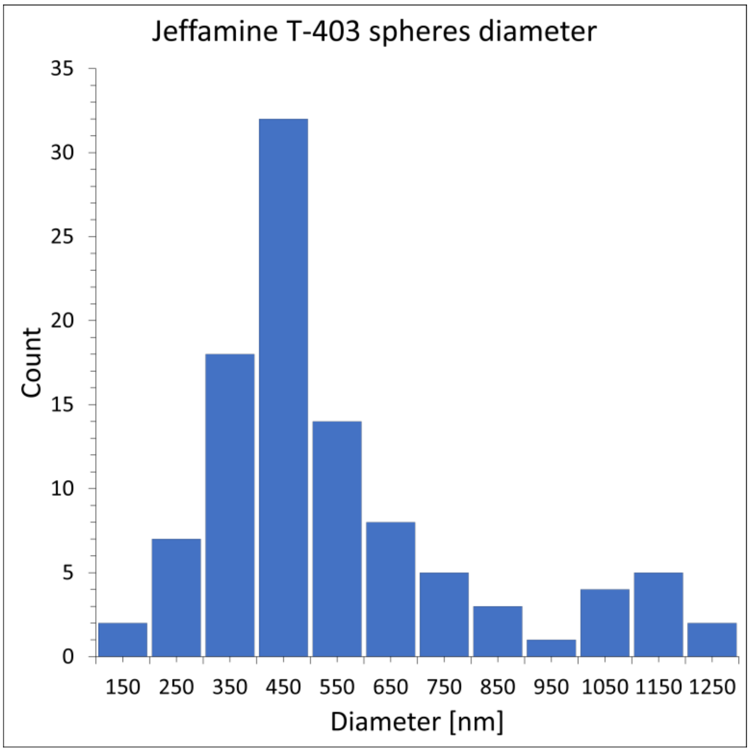

Figure S3: Size distribution of the nanospheres in crosslinked Jeffamine T403 microcylinders.

S4: Size distribution of the Jeffamine D-2000 segments and the gaps between them in Jeffamine D-2000 fibers

The length of the Jeffamine D-2000 segments and the gaps between them that are formed in the crosslinked Jeffamine D-2000 fibers were measured from CSLM images. A total of 40 segments and 40 gaps originated from four uncut long fibers were measured and the average length and distribution of both the segments and the gaps were calculated. The length of the segments was measured from edge to edge of the red emitted areas and the gaps were measured from edge to edge of the dark areas. The average length of the segments is 16.6±3.8 µm (**Figure S4A**), similar to the average length of the gaps which is 16.5±4.3 µm (**Figure S4B**).

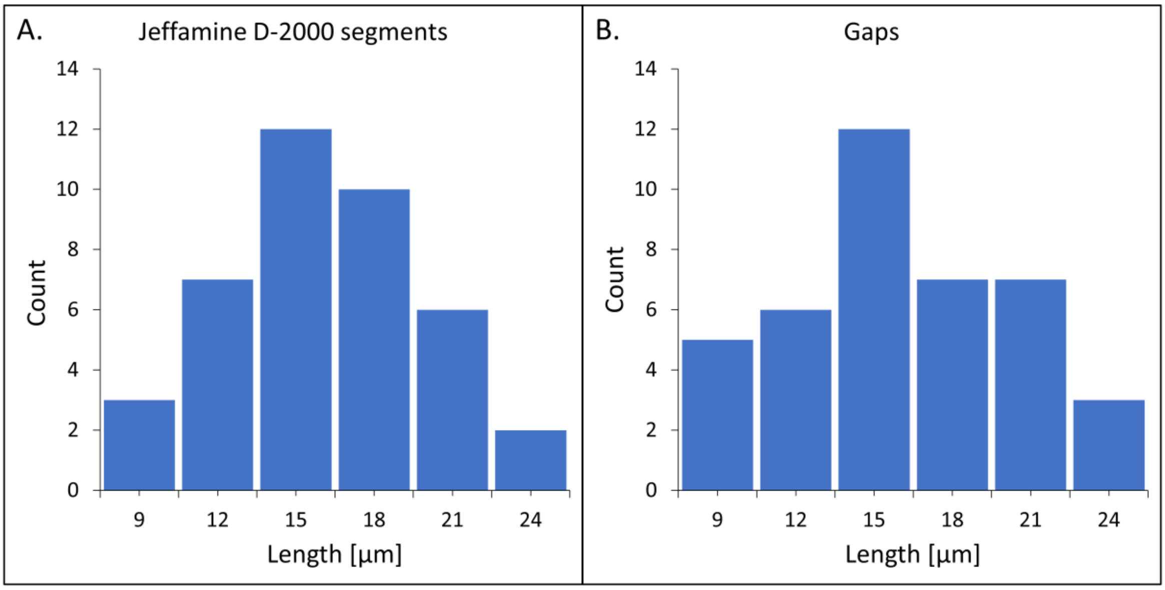

Figure S4: Size distribution of **A.** Jeffamine D-2000 segments and **B.** the gaps between the Jeffamine D-2000 segments of crosslinked Jeffamine D-2000 crosslinked fibers.

S5: Effect of GA concentration on the core's architecture

To examine the effect of the GA concentration in the collection gel on the inner morphology, Jeffamine T-403 fibers formed from the same spinning solution were collected into gels that contained different concentrations of GA. The gels were then dried, cryo-sectioned, and the formed microcylinders were thoroughly washed to remove the remaining gel. **Figure S5** shows a representative example of the microcylinders obtained for each GA concentration. In the case where no GA was added to the gel, the obtained fibers were hollow (**Figure S5A**), implying that the Jeffamine T-403 that was not crosslinked, dissolved in the water throughout the cleaning process and was washed away.

When the concentration of GA was 1% in the gel, hollow fibers with an inner layer of nanospheres were obtained (**Figure S5B**). This intermediate architecture indicates that the penetration of the GA occurs from the walls inward, and that it is relatively homogeneous along the fiber, strengthening the diffusion through the shell as a plausible mechanism. However, at a low concentration of GA, the crosslinking depletes the GA from the solution and hence the fiber was not fully filled with the spheres. Above 6% of GA in the gel, the obtained cylinders are fully filled with spheres (**Figure S5C and S5D**), suggesting that the concentration of GA is sufficient to form the full crosslinking of the amount of Jeffamine T-403 at the core. These results show that the architecture can be further controlled through the concentration of the ingredients of the systems, opening the path for highly porous hollow fiber, which are of potential for catalysis applications.

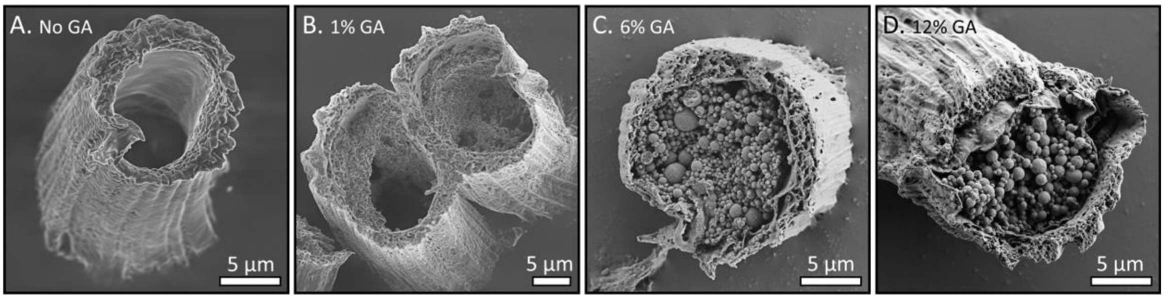

Figure S5: Cryo-sectioned Jeffamine T-403 fibers embedded into gels with different concentrations of GA: **A.** 0% GA, **B.** 1% GA, **C.** 6% GA, and **D.** 12% GA.

S6. Calculation of the mass of ruthenium adsorbed per length or volume units

The mass percentage of ruthenium adsorbed to the Jeffamine T-403 crosslinked fibers was measured by ICP-MS. Given the ruthenium mass percentage and the sectioned fiber's average diameter and length, the mass of ruthenium adsorbed per length or volume units of the fibers can be calculated.

The volume of a microcylinder (MC) was calculated according to a cylinder volume equation:

$$V_{MC} = \pi R^2 h_{MC} \qquad (S1)$$

where R is the outer radius of the fiber and h is the length of the microcylinder.

The mass of the ruthenium per one MC, m_{Ru} was calculated by:

$$m_{Ru} = \frac{M_{Ru}}{n_{MC}} \qquad (S2)$$

where M_{Ru} is the total mass of ruthenium in the sample, and n_{MC} is the number of MCs in the sample. From this, the ruthenium mass per length ($\overline{m_l}_{Ru}$) or its concentration in the fiber (C_{Ru}) can be estimated:

$$\overline{m_l}_{Ru} = \frac{m_{Ru}}{h_{MC}}, \qquad C_{Ru} = \frac{m_{Ru}}{V_{MC}} \qquad (S3,4)$$

The results obtained from the calculation are summarized in table S1.

Table S1: Calculation of the average concentration of ruthenium in a fiber

Number of sectioned MCs in the sample, n_{MC}*	8,800,000
Mass of the sample [mg]**	30
Mass of ruthenium in the sample, M_{Ru} [mg]**	3
Length of the MC, h_{MC} [µm]	50
Average outer diameter of the fibers ($2R$) [µm]	16.3
Volume of a MC, V_{MC} [mL]	1.0×10^{-8}
Ruthenium mass in a MC, m_{Ru} [mg]***	3.4×10^{-7}
**Ruthenium mass per length, $\overline{m_l}_{Ru}$, [µg/m]**	**6.8**
**Ruthenium concentration, C_{Ru} , [mg/mL]**	**33**

* The number of sectioned fibers per mL in the solution was calculated using a hemocytometer.

** As measured in the ICP-MS.

*** This is also the mass of ruthenium in 50 µm length and in 1×10^{-8} mL volume.

S7. Phase separation in the bulk solutions of polyamines and GA (50% in water)

As discussed in the manuscript, when examining the interface between a drop of Jeffamine T-403 and GA (50% in water), the formation of spontaneous emulsification is observed at the contact point, with Jeffamine T-403 dissolving into the GA phase, followed by a rapid formation of nano and microdroplets of the Jeffamine T-403 phase, which solidifies to form nano and microspheres (**Figure 1**). Because Jeffamine T-403 fully dissolves in water, we attribute the emulsification to the formation of crosslinked Jeffamine T-403 polymeric clusters, that continue to grow throughout the process. The growth leads to a significant reduction in the solubility, and hence results in phase separation.

When examining the same process with TEPA and GA, we observed a similar process, in which the first TEPA dissolves in the GA phase, and after a while, nano and microspheres are formed in the GA phase (**Figure S6 and movie S4**). Interestingly, the formation of spontaneous emulsification in the TEPA system is much slower than in the JeffamineT-403 system. This strengthen our assumption that the phase separation is related to the ongoing growth of a crosslinked polymer clusters, because the TEPA is smaller than Jeffamine T-403 and has more amine groups, hence it is more soluble. Consequently, the TEPA-GA polymer network must increase significantly in size to become insoluble, and therefore we see a delay in the starting time of phase separation process.

In the case of Jeffamine D-2000, the phase separation process is significantly different. Jeffamine D-2000 is much larger than the previous polyamine molecules, and has a long poly-propylene glycol backbone, which makes it is significantly less soluble in the GA-water phase. Hence, already upon the formation of contact, a clear boundary is observed between the Jeffamine D-2000 and the GA phases (**Figure S7 and movie S5**). At the same time, a vigorous formation of nano- and microdroplets is observed in the Jeffamine D-2000 phase, indicative of

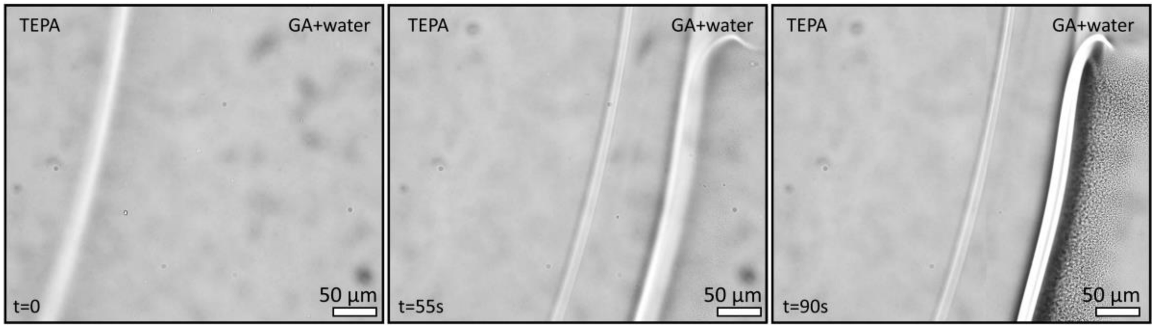

Figure S6: Time lapse showing the spontaneous emulsification of TEPA in the GA/water phase.

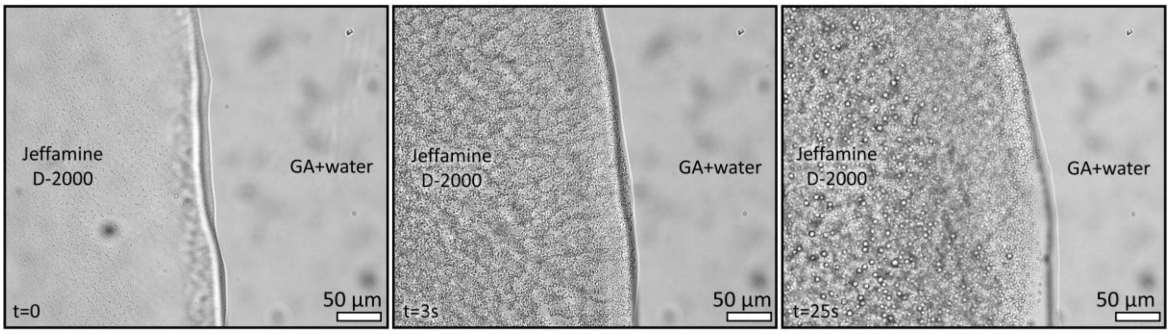

Figure S7: Time lapse showing the spontaneous emulsification of GA/water in the Jeffamine D-2000 phase.

the spontaneous formation of an emulsion of GA and water in the Jeffamine. As time progresses, the emulsion, which is confined to the Jeffamine-D2000 phase, becomes denser and eventually solidifies, taking the shape of the Jeffamine D-2000 phase. This behavior complies with the observed inner architecture in the Jeffamine D-2000 fibers, and that the shape of the solid segments is governed by the solidification and phase separation of the Jeffamine D-2000 inside the fiber, and not through emulsification, as is observed for the small polyamine molecules.
